# Cardiological Functional Assessment of National Olympic Team of Kazakhstan

**DOI:** 10.3390/jcm12247511

**Published:** 2023-12-05

**Authors:** Dauren Yerezhepov, Aidana Gabdulkayum, Galiya Bilyalova, Saya Amangeldikyzy, Ulan A. Kozhamkulov, Saule E. Rakhimova, Ulykbek Y. Kairov, Ainur Akilzhanova

**Affiliations:** 1 Laboratory of Genomic and Personalized Medicine, Center for Life Sciences, National Laboratory Astana, Nazarbayev University, Astana 010000, Kazakhstan; aidana.gabdulkayum@nu.edu.kz (A.G.); ulan.kozhamkulov@nu.edu.kz (U.A.K.); saule.rakhimova@nu.edu.kz (S.E.R.); 2 National Center for Sports Medicine and Rehabilitation, Almaty 020000, Kazakhstan; 3 Center for Sports Medicine and Rehabilitation, Astana 010000, Kazakhstan; 4 Laboratory of Bioinformatics and Systems Biology, Center for Life Sciences, National Laboratory Astana, Nazarbayev University, Astana 010000, Kazakhstan; ulykbek.kairov@nu.edu.kz

**Keywords:** athletes, cardiovascular evaluation, sudden cardiac death, risk management

## Abstract

Athletes carry an increased risk of cardiovascular (CV) conditions. Due to the relatively high loads and intensity of the training process, athletes’ CV systems undergo various adaptations, which can combine in the future and provoke unexpected outcomes. Most CV screening protocols have several successive steps. The aim of our study was to perform a cardiological functional assessment of the National Olympic Team of Kazakhstan via several noninvasive protocols to close the gaps between the approaches and collect solid data for the prevention of sudden cardiac death (SCD) incidence among Kazakhstani athletes. Methods: The methods used in this study were 12-lead resting electrocardiography (ECG), echocardiography, cardiointervalography, cardiopulmonary exercise testing (CPET), and HyperQ stress testing. Results: One case was detected via 12-lead resting ECG. Another case of the slowdown of the heart rate (HR) recovery was detected via cardiointervalography with no clinical signs and normal ECG. The HyperQ stress testing of the women’s basketball team detected a positive result in four leads in one athlete. Conclusion: Our results demonstrate that the CV systems of athletes require the implementation of several diagnostic methods in rest and stress conditions for more precise evaluation, with each of the methods fulfilling the whole picture for the prevention of such tragic events as sudden cardiac death and sudden cardiac arrest.

## 1. Introduction

Despite being some of the fittest individuals in society, athletes carry an increased risk of cardiovascular conditions [1]. Exercise is one of the most powerful tools for improving health and has been associated with beneficial changes in most cardiovascular risk factors, including lipids, blood pressure, insulin sensitivity, and weight [2,3,4,5]. But if moderate exercise (less than 4 h a week with low intensity) is considered a healthy activity that strengthens the cardiovascular system, high-intensity exercise for a long period can increase the risk for cardiovascular disorders (CVDs), sudden cardiac death (SCD), and sudden cardiac arrest (SCA) [6], with SCD being the leading cause of mortality in athletes during sports activity and exercise [7,8].

Pre-participation cardiovascular screening (PPCS) and systematic cardiovascular screening (SCVS) measures aim to identify and manage conditions associated with SCD and sudden cardiac arrest (SCA). The American Heart Association (AHA) recommends 14-point screening guidelines [9] combined with questionnaire and family history data [10]. The 12-lead resting electrocardiography (ECG) is the most accessible screening method for the majority of conditions and disorders associated with SCD, the main goal of which is the early detection of abnormalities [11,12]. ECG abnormalities can be found in most hypertrophic cardiomyopathy (HCM) patients. Also, EGC can diagnose ion channelopathies and ventricular preexcitation [13]. The physiological, structural, and electric adaptations of a highly trained athlete’s heart might have indicators similar to pathological conditions such as hypertrophic cardiomyopathy (HCM) or arrhythmogenic cardiomyopathy (ACM), and most of the SCD cases among athletes older than 35 are due to atherosclerotic coronary artery disease (CAD) [14]. This is where transthoracic echocardiography (TTE) comes into play to allow for a comprehensive initial visual evaluation of the cardiac structures and the changes in the response to exercise [15] and to distinguish the adaptations of an athlete’s heart (AH) from life-threatening pathologies [16]. TTE focuses on the characteristics and functions of the left ventricle (LV), left atrium, aorta, and right ventricle (RV), which also undergo structural and functional adaptations in highly trained athletes [17].

The 12-lead resting ECG gives the characteristics of the heart at rest, which can be used to detect abnormalities during normal conditions, and classic echocardiography allows taking very important measurements of the heart. The heart of an athlete undergoes different adaptations in response to continuous training and loads; thus, the behavior of the heart during loads or the training process gives valuable data for the evaluation of an athlete’s performance, working capacity, metabolism, level of adaptation, and abnormalities [18]. 

Heart rate variability (HRV) is an analysis of the variations in the intervals between heartbeats and has become a useful tool for the clinical investigation of fatigue, especially in athletes [19]. HRV may indicate a disease, because its reduction is associated with several risk factors including hypertension, diabetic neuropathy, and acute myocardial infarction [20]. HR recovery (HRR) is a very important indicator that provides valuable insight into the stress level. A delayed decrease in HR may indicate a lowering rate of vagal activity, which can lead to death [21]. 

Nowadays, the rapidly evolving landscape of diagnostics and screening makes it possible to develop new diagnostic methods or improve known ones and to combine different evaluation methods to obtain more data in a single test with the results already loaded into special software that can analyze many indicators and findings. One of the first methods developed was stress echocardiography, which, unlike classical TTE, is combined with physical, pharmacological, or electrical stress and is dedicated to the detection of many heart conditions, including myocardial ischemia and HCM [22,23]. 

Cardiopulmonary exercise testing (CPET) is an objective assessment that provides full data on the physiologic responses to exercise [24]. CPET provides valuable data on physiologic responses of several body systems (cardiorespiratory—lung, muscle, and cardiovascular systems) interconnected in tests and allows analyzing the body’s conditioning level and developing individual training programs for athletes [25]. 

Coronary artery disease (CAD) screening is largely based on exercise ECG testing, which has limited diagnostic accuracy, especially in women. Additionally, there are growing concerns over exposure to radiation and cutbacks in payment for costly cardiac imaging. HyperQ stress ECG testing provides a low-cost, noninvasive, radiation-free, and highly reliable CAD diagnostic solution [26]. 

PPCS and SCS aim to identify pathological conditions in athletes to decrease and/or prevent fatalities from devastating cardiovascular events, but there is no one-fits-all protocol [27]. Although 12-lead resting ECG is recommended by European guidelines, the American Heart Association (AHA) does not support 12-lead resting ECG screening in competitive athletes [28]. HRV-based screening and CPET are two of the last-line screening methods according to several guidelines [29,30].

The aim of our study was to perform a cardiological functional assessment of the National Olympic Team of Kazakhstan via several noninvasive protocols to the close gaps between approaches and collect solid data for the prevention of SCD incidence among Kazakhstani athletes.

## 2. Materials and Methods

### 2.1. Participants

Athletes who were included in the main and reserve National Olympic Team of Kazakhstan in various sports were recruited from June 2021 to March 2023. 

### 2.2. Cardiological Functional Assessment 

Physical evaluation and medical histories were evaluated via 14-point AHA guidelines. The 12-lead resting electrocardiography (ECG) was performed according to international guidelines, and results were analyzed according to the international criteria [7]. Transthoracic echocardiography (TTE) parameters, such as left ventricle (LV) dimension, global systolic and diastolic volume, stroke volume (SV) and stroke volume index (SVI), LV back wall thickness, and interventricular septum thickness, were analyzed. TTE was performed on an ACUSON S1000 Ultrasound System (Siemens, Erlangen, Germany). Cardiointervalography (EGC, cardiac rhythmography, and calculation of the HRV indicators) was performed on a POLY-SPECTRUM-8/EX software–hardware complex (SHC), version 1.1.1.22 (Neurosoft, Ivanovo, Russia), according to the international recommendations [28]. Cardiopulmonary exercise testing (CPET) was performed on a pulsar^®^ 3p (h/p/cosmos, Nussdorf, Traunstein, Germany). Speed, agility, and coordination were evaluated on a Speedzone (h/p/cosmos, Nussdorf, Traunstein, Germany). Motor cognitive and coordination skills were evaluated using a Witty SEM Reactive and Cognitive Training System (Microgate, Bolzano, Italy). HyperQ stress testing was performed on a CARDIOVIT CS-200 (Schiller, Baar, Switzerland). All tests were performed on an empty stomach between 8 a.m. and 10 a.m. Athletes were asked to skip morning training sessions to avoid deviations in CV indicators.

The study design of the present investigation was evaluated and approved by the Local Ethics Committee of the Institution and conforms to the ethical guidelines of the Declaration of Helsinki. All athletes included in this study were fully informed of the types and nature of the evaluations and signed the informed consent form.

### 2.3. Statistical Analysis

The continuous variables are summarized as mean and standard deviation (SD). The binary variables are shown as frequencies (*n*) and percentages (%). The Pearson chi-square test or Fisher’s exact test was used to compare the frequencies of the categorical variables, as appropriate. The Shapiro–Wilk test was used for the normal distribution testing. Data analysis was performed using SPSS 25.0 statistical software (IBM, New York, NY, USA).

## 3. Results

**Figure 1:**
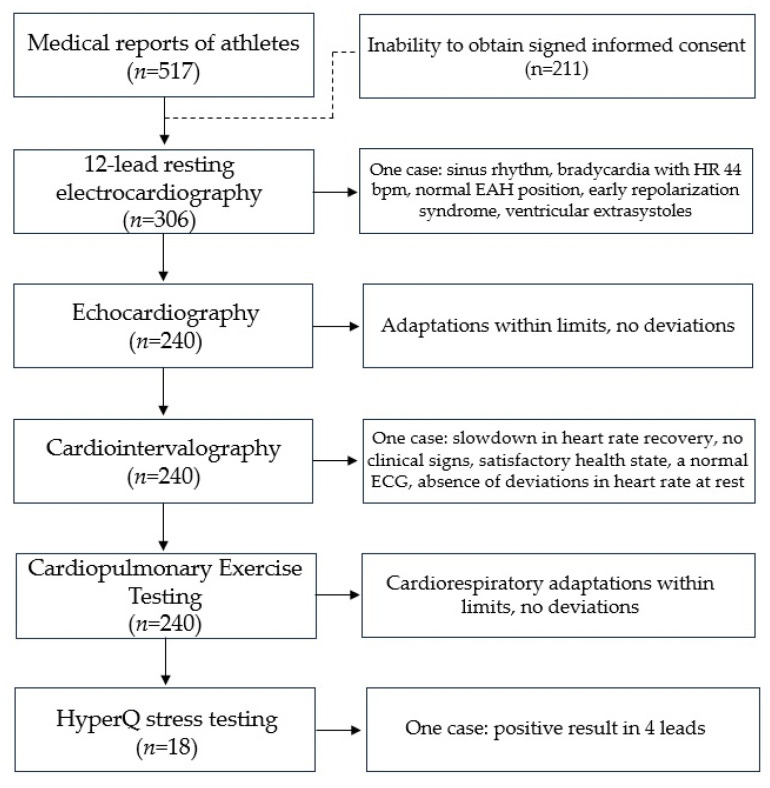
Study design and outcome of cardiological functional assessment.

Five hundred sixteen medical reports of athletes included in the Olympic Team of Kazakhstan in various sports were examined. Demographic data are shown in Table 1. The mean age was 21.9 ± 2.98 years, 340 (65.9%) were male, and about four-fifths were Asians (78.1%). Athletes had the rank of master of sports, master of sports of international class, and candidates for master of sports with at least five years of experience in sports. Athletes were primarily engaged in power sports (35%); less than one-fifth were engaged in cyclic sports (17.6%), and even fewer athletes were engaged in complex coordination sports and shooting (8.1% and 9.3%, respectively). Due to the inability to obtain or refusal to sign informed consent, the data of 211 individuals was excluded from the analysis of subsequent tests. Due to problems with the extraction of medical data from the database, we could obtain access to TTE, cardiointervalography, and CPET data of 240 athletes. HyperQ stress testing was performed to identify the presence of diagnostically significant signs of ischemia in women athletes that represent non-cyclic-load sport types. HyperQ stress testing could only be carried out at a specific medical organization where the device is located (CARDIOVIT CS-200). HyperQ stress testing was performed for 18 athletes (artistic gymnastics, *n* = 9, and basketball, *n* = 9). The study design and the outcome of the cardiological functional assessment are illustrated in Figure 1.

### 3.1. 12-Lead Resting EGC

Three hundred seven 12-lead resting ECG reports were analyzed. Considering that all athletes were members of the Olympic Team and had a high competitive level and many years of experience in sports activity, the number of individuals who had a normal resting ECG, according to the criteria described by Drezner J.A. et al. [7], was relatively small (104). In other cases, there were changes corresponding to the typical ones found in athletes, like sinus bradycardia, first-degree AV block, incomplete right bundle branch block (RBBB), early myocardial repolarization, and single extrasystoles (Table 2). Moreover, one athlete could have several of these changes.

However, it should be noted that the increase in sports experience leads to a rise in the number of registered ECG changes, including combined ones, which requires additional dynamic electrocardiographic monitoring during intensive training and competitive periods, despite the absence of violations during preventive examinations of athletes.

**Figure 2:**
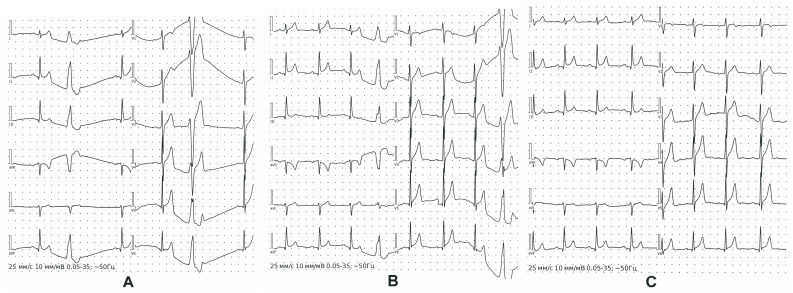
12-lead resting ECG of ventricular extrasystole case. Detection of the ventricular extrasystole for the first time (**A**); 12-lead resting ECG after revision of the training regimen and prescription of the cardio protectors (**B**); 12-lead resting ECG after radiofrequency ablation of the ectopic focus of extrasystole from under the right coronary cusp of the aortic valve (**C**).

As an example, we give a clinical observation of an athlete, master of sports, age 23, sport type boxing, sports experience 13 years, male, height 182 cm, weight 78 kg, BMI 23.5 kg/m^2^. Based on the results of the PPCS, the athlete was allowed to participate in sports. He suffered a mild viral infection. Within two months, in connection with the upcoming international competitions, he began intensive training (two sessions a day, 1.5 h each, six times a week). An unscheduled 12-lead resting ECG test recorded ventricular extrasystole for the first time during intensive preparation for competitions. The ECG indicators were as follows: sinus rhythm, bradycardia with HR 44 bpm, normal EAH position, early repolarization syndrome, and ventricular extrasystoles (Figure 2A). During 24 h Holter ECG monitoring in the background of sinus rhythm (min 54 beats per minute, max 118 beats per minute), the following indicators were recorded: PVCs—17,209; trigymenia—110 cases; pauses of more than 2s—2 (max—2666 ms). The training regimen was revised, and The World Anti-Doping Code-approved cardio protectors were prescribed. Control 12-lead resting ECG indicated AV conduction at the upper limit of normal ranges, slowing of intraatrial conduction, and nonspecific ST-T changes (Figure 2B). Twenty-four-hour Holter ECG monitoring revealed the following: PVCs—12,338 per day (single monomorphic, including trigeminy type 35; supraventricular extrasystole—3 per day, single); no ST depression. Echocardiography results were as follows: chamber sizes and wall thickness were within normal ranges, systolic and diastolic functions were not impaired. The PWC 170 test revealed a low level of physical performance (1332 kgm/min), a maximum oxygen consumption of 36 mL/min/kg, and a hypertensive-type blood pressure response. The athlete was removed from training and referred for consultation with an arrhythmology specialist. The diagnosis was cardiac arrhythmia and idiopathic ventricular extrasystole. An electrophysiological examination was performed, involving radiofrequency ablation of the ectopic focus of extrasystole from under the right coronary cusp of the aortic valve using the CARTO navigation system. After complete recovery, 12-lead resting ECG indicated the following: sinus rhythm, irregular; HRmax—71; HRmin—62; vertical position of the electrical axis of the heart; AV conduction at the upper limit of normal ranges; slowing of the intraatrial conduction; nonspecific ST-T changes (Figure 2C). Twenty-four-hour Holter ECG monitoring indicated the following: sinus rhythm; average HR 65 (min 43, max 115); PVCs—0; single supraventricular extrasystoles—5; pauses—0. Currently, the athlete continues to perform successfully.

### 3.2. Echocardiography

Echocardiography is a valuable method for the evaluation of an athlete since it provides information on cardiac morphology, function, and hemodynamics at a low cost [21,23]. The echocardiograms of 240 athletes were analyzed. The echocardiography analysis is shown in Table 3. 

All the athletes had approximately the same age, experience, and qualifications, which makes it possible to explain the differences in echocardiographic parameters via the specifics of their motor activity. The analysis of the echocardiography results is shown in Table 4.

The largest value of diastolic size of the LV (DSLV) is determined in representatives of the martial arts—5.6 cm. These values are significantly smaller in athletes in complex coordination sports—4.9 cm. Next come representatives of “endurance” and speed–strength sports. However, the increase in end-diastolic and end-systolic dimensions in representatives of different groups of motor activity is almost the same, and therefore, the stroke volume (SV) is greatest in the representatives of sports that develop endurance (96.7 mL), followed by martial arts (93.7 mL), team sports (93.6 mL), and speed–strength sports (93 mL).

Other indicators, such as LV posterior wall thickness (LV PWT) and the interventricular septal thickness (IST), turned out to be the biggest in athletes of the “endurance” group (1.3 cm and 1 cm, respectively). These values were smaller in team sports (0.99 cm and 1 cm, respectively). Next is the group of speed–strength sports, martial arts, the sixth group, and the group of complex coordination sports. However, considering the low weight characteristic of representatives of the complex coordination sports, their value of the LV PWT indicator was lower than that for endurance, speed–strength sports, and martial arts athletes.

The highest value of stroke index (SI) was found in the martial arts group (50.8 mL/m^2^), and the lowest value was found in representatives of the sixth group. At the same time, representatives of martial arts and complex coordination sports are characterized by the highest rates of contraction of the myocardium of the LV posterior wall and the lowest rates of relaxation. The calculated echocardiography indicators characterizing myocardial contractility are noteworthy. Representatives of the martial arts group have a significantly higher ejection fraction (EF) value than other groups (63%). This determines the large functional capacity of the LV at rest, which serves as a reserve of EF (the martial arts group in our studies consisted of Greco-Roman and freestyle wrestlers).

### 3.3. Cardiointervalography

Cardiointervalography quite effectively reflects the adaptive reactions of athletes’ bodies to intense training loads. HRV has several features depending on the type of sport, age, gender, and training cycle and provides invaluable information for assessing functional reserves, the correctness of the training process, moments of failure of adaptation, and entry into peak sports form [21]. The purpose of regular examination is not a comparative description of the performance of various athletes but the ability to identify early premorbid conditions and disorders of autonomic regulation. 

Analysis of cardiointervalography results (*n* = 240) showed an increase in the total activity of autonomic influences on the heart rhythm, a shift in the balance towards parasympathetic influences, and an increase in the degree of connection between the autonomous (segmental) levels of blood circulation regulation and the suprasegmental ones. Cardiointervalography of cyclic group showed higher values of maximum RR intervals (RRmax), standard deviation of NN intervals (SDNN), root mean square of successive differences between normal heartbeats or RR intervals (RMSSD), percentage of adjacent NN intervals that differ from each other by more than 50 ms (pNN50), coefficient of variation (CV), total power (TP), and high-frequency band (HF) and lower values of mode amplitude (AMo), vegetative balance index (VBI), and tension index (TI). These indicators show that these elite athletes have a new level of adaptation when sports results are achieved with less tension in the regulatory systems and an increase in the activity of the parasympathetic regulation link. 

Based on the data obtained from the spectral and temporal analysis of athletes, different degrees of activity of regulatory systems and individual characteristics of HRV were identified. The greatest differences among statistical indicators were recorded for RMSSD, SDNN, TP, HF, low-frequency band (LF), and very low frequency (VLF). Thus, the studied indicators made it possible to create a portrait of each athlete and monitor it throughout the entire macrocycle.

As an example, we give a clinical observation. The dynamic observation of this case is shown in Table 5. During several training camps, a boxer had good heart rate variability (HRV) indicators (Table 5, probes 1 and 2). After suffering a viral infection, the athlete did not show clinical signs, having a satisfactory health state, normal ECG, and an absence of deviations in heart rate (HR) at rest. During the training session, a slight slowdown in heart rate recovery (HRR) was recorded (Polar-team 2 device). Additional examinations were conducted (Table 5, probes 3 and 4). Control indicators after changes in loads in the training process and pharmacological support showed normalization of HRR (Table 5, probes 5 and 6). 

It should be noted that traditional methods of physiological research (HR and BP) did not reveal a decrease in the adaptive and functional capabilities of the body, but with an additional examination (probe 3), a sharp reduction in parasympathetic influence was revealed: a decrease in RMSSD, SDNN, TP, HF, and LF; and an increase in VLF and AMo; and a significant increase in the tension index (TI). This means that the rhythmograms and the structure of the HRV indicated a pathological stabilization of the HR modulation with a transition of its regulation from the reflex or vegetative level to a lower humoral-metabolic level, which cannot ensure quick homeostasis. The state of neurohumoral regulation was characterized by a low level of vagal, sympathetic, and humoral-metabolic (cerebral, ergotropic) influences in the modulation of the heart rate.

These examinations confirmed a significant decrease in the functional state, overexertion, and autonomic dysfunction, meaning that physical activity at that time did not correspond to the boxer’s functional capabilities. The coaching council developed an individual training plan, monitored compliance with the work and rest regime, and carried out a pharmacological correction, and an improvement in performance in the fourth examination (after three weeks) was noted. Accordingly, positive dynamics were noted during subsequent follow-up examinations (Table 5, probes 5 and 6). After full recovery, the athlete began training as usual, resulting in a successful competition performance.

### 3.4. Cardiopulmonary Exercise Testing (CPET)

Cardiopulmonary exercise testing (CPET) plays a special role in sports medicine. CPET is the only method for assessing the aerobic capacity of athletes. The main goal is to analyze the maximum oxygen consumption to evaluate the professional level of the athlete. During stress testing with a gas analyzer, the anaerobic threshold is also determined to identify the reserve capabilities of the athlete’s body [24].

The CPET results of 240 athletes are shown in Table 6. High PWC indicators in cyclic sports show a high level of physical performance. These indicators show, first, the effective performance of the cardiorespiratory system, which is due to the specifics of the sport: the cyclical nature and the development of endurance. The functional level of members of Olympic teams is characterized by the highest values of work and power of the loads performed, which are associated with high aerobic abilities, manifested both in high oxygen consumption per unit of body weight and load power and in later achievement of the threshold level of anaerobic metabolism. A high level of VO2max is one of the prerequisites for an athlete to achieve a highly competitive level but does not predetermine his or her unconditional success.

In power sports, most cases showed a moderately normotonic type of pressure response to exercise (SBP 167.4 ± 21.64, DBP 88.33 ± 10.33). In five cases, there was a hypertonic type of reaction, and in one case, there was a hypotonic type of reaction. The normotonic type of reaction is the most favorable and reflects good adaptation to physical activity. Physical exercise tolerance (indicated by MET) 10.25 ± 1.42 is very high, and VO2max indicators meet the standards for power sports. All indicators of team sports and complex coordination sports were in normal ranges according to sport type.

### 3.5. HyperQ Stress Testing

HyperQ stress testing identifies factors and the degree of risk of organic myocardial damage. The test was performed according to the typical ramp protocol (25 W/min ramp) [26]. The HyperQ indicator was processed using the Schiller HyperQ software package (version V3). Due to the small number of participants, deep analytical processing was excluded from the scope of the current work. Eighteen athletes of the non-cyclic-load sports type were taken for the test. Data are shown in Table 7.

The average age of the representatives of rhythmic gymnastics was 18 years (excluding months), and that of the representatives of basketball was 26 years (which is probably due to the specifics of the sport). All subjects had more than 10 years of experience. No one had any complaints regarding the cardiovascular system. At the same time, indications in the anamnesis of changes in previous ECGs (athletic heart) were higher in the younger cohort.

Each athlete underwent 12-lead resting ECG, ECG testing under stress, and HyperQ software (version V3) processing. 12-lead resting ECG indicated (Table 8) the presence of adaptations like sinus bradycardia, pacemaker migrations, atrial or ventricular extrasystole, atrioventricular (AV) block of the I-II degrees, high voltage of the QRS (HV-QRS), changes in the T wave in the inferolateral regions (reflecting hypertrophy of LV), deep inverted T waves in the anterolateral leads, incomplete RBBB, and other arrhythmias. 

12-lead resting ECG showed that the most common are bundle branch blocks (incomplete), sinus bradycardia, and nonspecific ST changes, which were recorded more in representatives of artistic gymnastics.

ECG stress tests were directed to obtain data for the presence of diagnostically significant signs of ischemia, verification of arrhythmia (if present), level of exercise tolerance (ET) according to the METS indicator, BP response at different stages of the test, and recovery indicators. The ECG stress test results are shown in Table 9. 

According to ECG stress test results, the ET METS indicator for the artistic gymnastics team was better by 1.4 units, which is probably due to the younger age of the artistic gymnastics team. Recovery rates and hypertensive BP response to stress were also slightly higher in female basketball players.

The Schiller HyperQ software package (version V3) was used to perform an assessment for the presence of negative reduction ≥ 50%, absolute reduction ≥ 1 μV, and HyperQ positive tests in three or more positive leads. The results of the HyperQ stress test are shown in Table 10.

HyperQ positive lead results were prevalent for the basketball team. There were no positive test results in the cohort of artistic gymnastics. At the same time, among the basketball players, one athlete had a positive result in four leads, which was reported to the team doctor and the head coach. All female athletes with positive leads had a history of “athlete’s heart” in anamnesis, meaning the HyperQ stress test can be considered sensitive for use in sports medicine.

## 4. Discussion

Sudden cardiac death (SCD) is the most common cause of death among athletes who engage in strenuous physical activity [7]. The precise incidence rate of such devastating tragedies is challenging to determine due to methodological differences and the heterogeneous nature of the study populations. Current studies show an incidence of SCD in athletes ranging between 0.5 and 13 deaths in 100,000. The majority of deaths happen in older athletes (≥35 years), affect more men than women, and occur during or immediately after exercise. The nature of SCD occurrence suggests that exercise might be a trigger in predisposed athletes, and up to 80% of SCD events occur without evaluable signs [8,31]. 

The cheapest and most accessible screening method recommended by the American Heart Association is a health questionnaire and physical examination [7], which has poor sensitivity because most athletes are asymptomatic prior to death [32]. The 12-lead resting ECG remains the most effective and widely used strategy for screening for cardiovascular disease in athletes [7]. American Heart Association (AHA) does not support CV screening if symptomatic (chest pain, dyspnea, palpitations, etc.) CV risk is not registered, while European guidelines recommend performing 12-lead resting ECG systematically [30,33]. The longer an athlete trains, the more ECG findings, including combined ones, might rise, especially in older athletes. Findings such as sinus bradycardia and first-degree AV block can be considered compensatory, associated with high myocardial contractility, as evidenced by the ability of such athletes to demonstrate a high level of performance, but as more combined indicators arise, more evaluation is needed. The number of false-positive tests is one of the main concerns about ECG screening in athletes, which has led to the implementation of several improvements in ECG interpretation criteria in the last ten or twenty years based on the age and ethnicity of an athlete and type and intensity of sports discipline [7,34]. Development and improvement of ECG interpretation criteria have led to a decrease in false positive results, but limitations are still present [35]. The extrasystole case in our study is a condition that can be easily detected by ECG. In this case, ECG achieved its main goal—early detection of abnormality. The early detection of such conditions and timed application of preventative measurements resulted in the complete recovery of the athlete considered as a case.

The CV system of any athlete can result in substantial adaptations for regular and long periods of physical activity to increase the physical performance of the athlete. The results of 12-lead resting ECG were drawn on paper, and the behavior of lines demonstrated changes in electrical activities. The development of echocardiography revolutionized screening and gave the opportunity to visualize these adaptive changes in cardiac structures. Adaptations lead to morphological, functional, and regulatory changes in an athlete’s heart. Changes may be characterized by increased mass, cavity dimensions, and wall thickness with at least normal systolic and diastolic function [9]. The remodeling of an athlete’s heart depends on various factors such as the type of exercise and duration, and changes might manifest in some pathological conditions, such as ACM or HCM; any sports doctor or cardiology specialist must consider any possible scenario [36]. Echocardiography made it possible to perform very important measurements of these changes precisely and interpret them accordingly to distinguish the remodeling from life-threatening pathologies [16], which was the trigger for multiple clinical investigations and subsequential development of recommendations, screening strategies, and distinguishing criteria for proper interpretation of echocardiography results in athletes [37]. 

Echocardiography is recommended to exclude structural heart conditions associated with SCD that cannot be detected by ECG, especially mitral valve prolapse, coronary artery anomalies, bicuspid aortic valve, and dilatation of the aorta, and then monitor the cardiac remodeling response to exercise for late cardiomyopathies and wall motion abnormalities provoked by CAD or myocarditis [38]. As for ECG, echocardiography has main limitations linked to age, sex, and ethnicity. In addition, heart size is dependent on body size, and echocardiography indicators and structural indices must be scaled according to a relevant body size scalar [39]. Many research works showed that adaptations of different parts of an athlete’s heart have different levels in different populations across the world and thus have different “normal limits” in the same sports type [17]. Age may lead to an increase in the number of adaptations, which can combine to switch an athlete to a higher level and/or increase the risk of cardiological conditions. This makes distinguishing adaptive changes in cardiac structures in response to exercise from mimicking cardiac pathologies difficult and requires a combination of ECG results, 24 h Holter monitoring, cardiac magnetic resonance imaging (MRI), and stress tests. The analysis of echocardiograms of athletes recruited in our research was performed according to international recommendations. All the findings revealed adaptive changes, and they were within the normal range limits of certain sport types. 

Screening methods at rest, like resting ECG and echocardiography, are sources of valuable indicators required for diagnosing cardiac abnormalities, but stress tests give information about how the heart behaves during load [31]. This information opens access to the capabilities of an athlete’s heart under physical load and, most importantly, can identify abnormalities that cannot be detected at rest, notably in asymptomatic adults [18]. Many studies showed positive results for many cardiac disorders by exercise testing for asymptomatic individuals, but the low prevalence of the disease is the main limitation of the test [39]. 

HRV is a noninvasive method that indicates acute fatigue and responses to physical demands. It has several features depending on the type of sport, age, gender, and training cycle and is invaluable information for assessing functional reserves, the correctness of the training process, moments of failure of adaptation, and entry into peak sports form [21]. Beat-by-beat fluctuations in the cardiac rhythm observed over a given period are the basis of HRV analysis and are considered as a reflection of the autonomic nervous system (ANS) [40]. HRV measurement of an athlete in resting conditions is recommended to be performed for 30 or 5 min because long monitoring times, such as 24 h, will give very extensive fluctuations due to differences in daily physical activity [41]. The normal limits of HRV, like in ECG and echocardiography, vary with age, sex, ethnicity, and sport type. In addition, long-term training may lead to increased HRV in athletes, especially with the presence of a strong aerobic component [42]. 

After achieving peak workload, athletes spend several minutes in a cool-down period (called a recovery period) during a stress test. HRR is a powerful independent prognostic marker that is used for the prescription and monitoring of an athlete’s training and depends on fatigue, type of exercise, exercise mode, and age [43]. A reduction in slowdown in HRR is a strong predictor of cardiovascular events leading to death [44]. The case presented in our study indicates the importance of HRR detection since the athlete had no clinical signs and normal ECG indicators with the absence of deviations in HR at rest. Again, timed utilization of screening methods saved not only the career but probably the life of this athlete. 

HRV testing (cardiointervalography) and CPET are very valuable testing methods and are used to evaluate cardiac indicators; while the former evaluates adaptive reactions to intense training loads by different changes in HRV, the latter evaluates the aerobic capacity of the organism. Still, there are no versatile standards for the measurements of adaptation to physical activity due to limitations of age, gender, ethnicity, and sports disciplines. In our study, CPET results indicated a high level of adaptations of the athletes in corresponding sport types. 

New methods of in-depth ECG analysis such as high-frequency QRS (HF-QRS) and HyperQ are relevant. Studies of changes in HF-QRS in various clinical conditions have demonstrated a close connection between HF-QRS and myocardial ischemia. Myocardial ischemia correlates with decreased HF-QRS intensity at rest and during a stress test. During HyperQ stress testing of the women’s basketball team in our study, one athlete had a positive result in four leads. The athlete was dropped from the team and was directed to cardiac evaluation.

Our results demonstrate that the CV system of athletes requires several diagnostic methods in rest and stress for more precise evaluation, with each of the methods fulfilling the whole picture for the prevention of such tragic events as SCD and SCA. 

### Limitations

Some limitations of the present study should be acknowledged. First, there is an uneven representation of athletes in the different sports categories, with some being underrepresented (e.g., power sports vs. team sports). Second, all athletes spend much more time in training than in competition, and they had different loads according to their age, weight, and experience in the same sport type during training camps prior to tests. Finally, we acknowledge that the addition of the results of a positive control group comprising non-athletic healthy subjects could provide some data, but this was not the purpose of this study since only athletes who were included in the main and reserve National Olympic Team of Kazakhstan in various sports were recruited.

## 5. Conclusions

Our results demonstrate that the CV system of athletes requires the implementation of several diagnostic methods in rest and stress conditions for more precise evaluation, with each of the methods fulfilling the whole picture for the prevention of such tragic events as sudden cardiac death and sudden cardiac arrest.

## Figures and Tables

**Table 1:** Demographic data (*n* = 516).

Age (years)		21.9 ± 2.98 (18–35)
Gender, *n* (%)		
Male/Female		340 (65.9)/176 (34.1)
Nationality, *n* (%)		
Asian/Caucasian		403 (78.1)/113 (21.9)
Years of training (years)		15 (5–21) *
Hours of training/week (h/week)		23 (18–28) *
Weight, kg		80.56 ± 24.33
Height, cm		175.21 ± 9.56
BMI, kg/m^2^		25.75 ± 6.78
SBP, mmHg		113.75 ± 12.46
DBP, mmHg		72.28 ± 10.42
HR, bpm		58.61 ± 11.23
Sport types		
	Power Sports (Greco-Roman and freestyle wrestling, judo, taekwondo, boxing, weightlifting, karate **)	181
	Cyclic Sports (road cycling, kayaking, open water swimming, biathlon, Nordic combined, triathlon, rowing slalom, synchronized swimming)	91
	Complex Coordination Sports (rhythmic and artistic gymnastics, trampolining, figure skating, alpine skiing)	42
	Shooting (shooting, clay pigeon shooting, and archery)	48
	Team Sports (women’s handball, rugby)	28
	Other Sports (fencing (epee and saber), mountain biking, snowboarding, ski jumping, pentathlon, rock climbing, curling, equestrianism)	174

* Median (25th–75th percentiles); ** was excluded from Olympic program after Tokyo 2020; BMI: body mass index; DBP: diastolic blood pressure; HR: heart rate; SBP: systolic blood pressure.

**Table 2:** Frequency and structure of changes on the ECG (*n* = 307).

Changes on ECG	Type of Sports			
	Cyclic (*n* = 91)	Power (*n* = 146)	Team (*n* = 28)	Complex Coordination (*n* = 42)
Sinus bradycardia	10 (10.9%)	26 (17.8%)	3 (10.7%)	3 (7.1%)
Sinus tachycardia	5 (5.5%)	5 (3.4%)	1 (3.6%)	2 (4.8%)
Extrasystole	4 (4.4%)	13 (8,9%)	2 (7.1%)	3 (7.1%)
Pacemaker migration and atrial rhythm	9 (9.9%)	7 (4.8%)	2 (7.1%)	2 (4.8%)
First-degree AV block	3 (3.3%)	9 (6.2%)	1 (3.6%)	2 (4.8%)
RBBB	10 (10.9%)	16 (10.9%)	3 (10.7%)	5 (11.9%)
Early myocardial repolarization	14 (15.4%)	23 (15.7%)	4 (14.3%)	6 (14.2%)
Impaired repolarization of PWLV	2 (2.2%)	6 (4.1%)	1 (3.6%)	1 (2.4%)

AV: atrioventricular; PWLV: posterior wall of left ventricle; RBBB: right bundle branch block.

**Table 3:** Echocardiography findings.

Indicator	Cyclic Sports (*n* = 71)	Power Sports (*n* = 114)	Team Sports (*n* = 22)	Complex Coordination Sports (*n* = 33)
HR, bpm	57.28 ± 0.83	59.38 ± 0.93	56.44 ± 0.75	55.42 ± 0.71
SBP, mmHg	114.0 ± 0.69	117.0 ± 0.75	116.0 ± 0.63	115.0 ± 0.66
DBP, mmHg	77.1 ± 0.64	78.1 ± 0.74	77.1 ± 0.68	77.0 ± 0.67
BSA, m^2^	1.66 ± 0.07	1.96 ± 0.08	1.76 ± 0.06	1.55 ± 0.04
Aorta, mm	2.39 ± 0.09	2.44 ± 0.08	2.41 ± 0.07	2.22 ± 0.05
EDS LV, mm	5.42 ± 0.03	4.92 ± 0.11	5.39 ±0.08	4.88 ± 0.06
ESS LV, mm	3.51 ± 0.07	3.48 ± 0.09	3.50 ± 0.06	3.49 ± 0.07
EDSI LV, mm/cm^2^	24.48 ± 0.94	26.25 ± 0.90	23.52 ± 0.92	27.22 ± 0.91
EDVI LV, mL/m^2^	61.33 ± 2.69	63.50 ± 3.01	62.34 ± 2.81	63.23 ± 2.62
LA, cm	3.61 ± 0.09	2.97 ± 0.11	3.48 ± 0.10	3.28 ± 0.09
LA volume, mL	44.50 ± 1.94	35.50 ± 1.94	34.45 ± 2.06	25.43 ± 2.08
SV, mL	88.80 ± 3.39	71.80 ± 3.39	68.99 ± 3.42	66.84 ± 3.22
EDV, mL	147.60 ± 2.62	121.60 ± 5.90	121.56 ± 5.99	108.45 ± 5.66
ESV, mL	49.67 ± 3.11	42.21 ± 3.11	39.85 ± 3.14	41.66 ± 3.10
EF, %	63.67 ± 1.15	62.67 ± 1.15	61.22 ± 1.19	62.21 ± 1.15
LV PWT, cm	1.2 ± 0.02	0.99 ± 0.02	0.97 ± 0.02	0.94 ± 0.01
LV IST, cm	1.1 ± 0.01	0.99 ± 0.03	1.0 ± 0.01	1.0 ± 0.01
LV MM, g	191.60 ± 13.37	180.60 ± 12.38	176.58 ± 12.42	168.22 ± 11.24
TAPSE, cm	1.79 ± 0.02	1.86 ± 0.02	1.76 ± 0.02	2.00 ± 0.02
LV MM index, g/m^2^	97.53 ± 3.14	97.13 ± 3.12	95.42 ± 3.19	94.38 ± 3.11
LV RWTI	0.43 ± 0.01	0.40 ± 0.01	0.39 ± 0.01	0.38 ± 0.01

BSA: body surface area; DBP: diastolic blood pressure; EDS: end-diastolic size; EDSI: end-diastolic size index; EDV: end-diastolic volume; EDVI: end-diastolic volume index; EF: ejection fraction; ESS: end-systolic size; ESV: end-systolic volume; HR: heart rate; IST: interventricular septal thickness; LA: left atrium; LV: left ventricle; LV MM: left ventricular myocardial mass; LV RWTI: relative wall thickness index; PWT: posterior wall thickness; TAPSE: tricuspid annular plane systolic excursion; SBP: systolic blood pressure; SV: stroke volume.

**Table 4:** Analysis of echocardiography results.

Group	Diastolic Size of LV, cm	End- Diastolic Volume, mL	End- Systolic Volume, mL	Stroke Volume, mL	Stroke Index, mL/m^2^	LV Posterior Wall Thickness, cm	Interven-Tricular Septal Thickness, cm
1	5.5 ± 0.03	148.8 ± 2.62	50.7 ± 0.9	96.7 ± 1.37	50.8 ± 0.62	1.3 ± 0.02	1 ± 0.01
2	5.3 ± 0.02	141.8 ± 2.36	49.2 ± 1.23	93.6 ± 1.46	49.6 ± 0.59	0.99 ± 0.01	1 ± 0.02
3	5.4 ± 0.09	142.9 ± 4.5	49.8 ± 3.02	93 ± 3.4	48.7 ± 0.37	1.3 ± 0.03	0.9 ± 0.02
4	5.6 ± 0.03	155.8 ± 2.6	51.3 ± 0.89	93.7 ± 1.7	51 ± 0.75	1.3 ± 0.03	1.2 ± 0.01
5	4.9 ± 0.04	143.6 ± 2.33	50.2 ± 0.97	78.7 ± 1.36	50.6 ± 0.62	1.3 ± 0.2	0.9 ± 0.1
6	5.2 ± 0.03	138.4 ± 2.62	48.5 ± 0.33	79.8 ± 1.36	48.1 ± 0.77	1.2 ± 0.04	1 ± 0.02

Group 1: endurance sports; group 2: team sports; group 3: speed–strength sports; group 4: martial arts; group 5: complex coordination sports; group 6: sport types that were not included in any of the other groups; LV: left ventricle.

**Table 5:** Dynamic observation of the case.

**Parameter**	**Background**					
	**Probe 1**	**Probe 2**	**Probe 3**	**Probe 4**	**Probe 5**	**Probe 6**
Mo, s	1.05	1.08	0.687	1.02	1.2	1.08
AMo, %	25.4	17.3	79.7	52.9	25.4	20.6
HR, s	0.51	0.582	0.215	0.207	0.583	0.515
VBI, units	49.8	29.7	371	255	43.6	39.9
RPAI, units	24.1	23.2	87.1	44.1	24.9	19.1
VRI, units	1.86	1.59	6.77	4.03	1.68	1.8
TI, units	23.6	13.8	270	164	20	18.5
	**Orthostatic**					
Mo, s	0.737	0.762	0.525	0.776	0.648	0.761
AMo, %	41.2	35.1	77.6	52.8	35	34
HR, s	0.34	0.454	0.109	0.214	0.377	0.368
VBI, units	121	77.3	512	247	92.9	92.3
RPAI, units	55.9	46.1	98.2	81.5	45.1	44.6
VRI, units	3.99	2.89	17.5	7.21	3.42	3.57
TI, units	82.2	50.7	678	190	59.9	60.7

TI: tension index; VBI: vegetative balance index; RPAI: regulatory processes adequacy indicator; VRI: vegetative rhythm index.

**Table 6:** Stress test results (*n* = 240).

Indicator	Cyclic Sports (*n* = 71)	Power Sports (*n* = 114)	Team Sports (*n* = 22)	Complex Coordination Sports (*n* = 33)
SBP max, mmHg	172.0 ± 13.33	167.4 ± 21.64	166.0 ± 12.25	168.2 ± 11.21
DBP max, mmHg	55.1 ± 11.18	88.33 ± 10.33	57.1 ± 12.22	55.8 ± 10.88
HR max, bpm	154.4 ± 9.2	152.0 ± 21.62	151.4 ± 8.9	151.1 ± 8.7
MET, kcal/min	14.16 ± 1.38	10.25 ± 1.42	6.52 ± 1.50	6.10 ± 0.62
PWC absolute, kg/m	2171.75 ± 201.82	1399.75 ± 209.92	1983.06 ± 214.16	1898.66 ± 213.46
PWC relative	33.65 ± 4.83	52.69 ± 12.15	32.42 ± 4.65	31.60 ± 5.03
VO2max absolute, mL/min	5714.25 ± 846.5	4019 ± 301.8	4256.25 ± 342.6	2440.2 ± 67.6
VO2max relative, mL/kg/min	76.25 ± 14.38	52.78 ± 13.16	53.20 ± 10.22	47.2 ± 6.9

MET: the metabolic equivalent of the task; PWC: physical working capacity; VO2max: maximum oxygen consumption.

**Table 7:** Data of athletes that were taken for HyperQ stress testing (*n* = 18).

Sport Type	*n*	Age (Mean)	Experience	Complaints for “Heart”	“Athlete’s Heart”, *n* (%)
Artistic gymnastics	9	18	>10	No	7 (77)
Basketball	9	26	>10	No	5 (55)

**Table 8:** 12-lead resting ECG indicators of artistic gymnastics and basketball teams (*n* = 18).

Adaptations	Artistic Gymnastics	Basketball
Bundle branch blocks	6	4
Nonspecific ST changes	5	3
Sinus bradycardia	6	3
Left ventricle hypertrophy	-	2
Rhythm disturbances	1	1
Total	7	5

**Table 9:** ECG stress test results (*n* = 18).

Sport Type	ET METS	Slow Recovery	Hypertensive BP Reaction	Nonspecific ECG Changes
Artistic gymnastics	11.5	2	-	7
Basketball	10.1	4	2	5

ET: exercise tolerance; METS: metabolic equivalents.

**Table 10:** HyperQ analysis results for positive leads (*n* = 18).

Sport Type	Positive Leads 1–3	Positive Leads 3 or More
Artistic gymnastics	4	-
Basketball	6	1

## Data Availability

The data presented in this study are available on request from the corresponding author. The data are not publicly available due to ethical and privacy restrictions indicated in informed consent.
